# American ginseng acutely regulates contractile function of rat heart

**DOI:** 10.3389/fphar.2014.00043

**Published:** 2014-03-14

**Authors:** Mao Jiang, Juan M. Murias, Tom Chrones, Stephen M. Sims, Edmund Lui, Earl G. Noble

**Affiliations:** ^1^School of Kinesiology, Faculty of Health Sciences, Western UniversityLondon, ON, Canada; ^2^Ontario Ginseng Innovation and Research ConsortiumLondon, ON, Canada; ^3^Department of Physiology and Pharmacology, Western UniversityLondon, ON, Canada

**Keywords:** heart rate, developed pressure, Langendorff perfused heart, ginsenoside, cardiac myocyte

## Abstract

Chronic ginseng treatments have been purported to improve cardiac performance. However reports of acute administration of ginseng on cardiovascular function remain controversial and potential mechanisms are not clear. In this study, we examined the effects of acute North American ginseng (*Panax quinquefolius*) administration on rat cardiac contractile function by using electrocardiogram (ECG), non-invasive blood pressure (BP) measurement, and Langendorff isolated, spontaneously beating, perfused heart measurements (LP). Eight-week old male Sprague–Dawley rats (*n* = 8 per group) were gavaged with a single dose of water-soluble American ginseng at 300 mg/kg body weight. Heart rate (HR) and BP were measured prior to and at 1 and 24 h after gavaging (ECG and BP). Additional groups were used for each time point for Langendorff measurements. HR was significantly decreased (ECG: 1 h: 6 ± 0.2%, 24 h: 8 ± 0.3%; BP: 1 h: 8.8 ± 0.2%, 24 h: 13 ± 0.4% and LP: 1 h: 22 ± 0.4%, 24 h: 19 ± 0.4%) in rats treated with water-soluble ginseng compared with pre or control measures. An initial marked decrease in left ventricular developed pressure was observed in LP hearts but BP changes were not observed in BP group. A direct inhibitory effect of North American ginseng was observed on cardiac contractile function in LP rats and on fluorescence measurement of intracellular calcium transient in freshly isolated cardiac myocytes when exposed to ginseng (1 and 10 μg/ml). Collectively these data present evidence of depressed cardiac contractile function by acute administration of North American ginseng in rat. This acute reduction in cardiac contractile function appears to be intrinsic to the myocardium.

## INTRODUCTION

Ginseng is a popular herbal remedy widely used to improve cardiac health and circulation. Chronic *Panax* *ginseng* treatment has been shown to improve cardiac performance, as reflected by improvements in isolated heart contractility and mitochondrial oxidation (Toh, 1994; Chen, 1996), as well as to increase protection against myocardial ischemia/reperfusion damage by enhancing NO release (Chen, 1996).

In human research, the effects of different herbal compounds, containing ginseng products, on the cardiovascular system are equivocal. On one hand, supplements containing ginseng have been found to decrease heart rate (HR), suggesting that ginseng may affect the autonomic nervous system (Kiesewetter et al., 1992; Zheng and Moritani, 2008). On the other hand, the effects of ginseng on BP remain controversial. For instance, increases (Siegel, 1980), decreases (Kiesewetter et al., 1992; Han et al., 1998), and no change (Stavro et al., 2005, 2006) in BP have been reported. The reasons for these discrepancies might be related to the postulated biphasic actions of ginsenosides on BP, with initial decreases, followed by increases (Kaku et al., 1975; Chen et al., 1984). Additionally, the effects of chronic versus acute supplementation with ginseng have to be considered. For example, although chronic ginseng treatment appears to improve cardiac performance, the effects of acute administration of ginseng on cardiovascular function remains controversial (Bahrke and Morgan, 1994; Gillis, 1997) as enhanced (Toh, 1994), little change or depressed cardiac function (Jin and Liu, 1994; Jin, 1996), have been reported.

Another factor modulating the effects of ginseng is the type of ginseng used during the intervention. It has been recognized that North American ginseng (*P. quinquefolius*) differs from other species of ginseng like Asian ginseng (*P. ginseng*) in its active ingredients and characteristic biological responses. North American ginseng has a ginsenoside profile different from that of Asian ginseng in terms of total ginsenosides, the ratio of protopanaxadiol (PPD) to protopanaxatriol (PPT), and other marker ginsenosides (Qi et al., 2011). For example, ginsenosides Rb_1_ and Re appear to be most abundant in North American ginseng, whereas Asian ginseng (Chinese/Korean Ginseng) is enriched in Rg1 and Rg2 ginsenosides. Traditional Chinese medicine asserts that North American ginseng promotes *yin* in the body while Asian ginseng promotes *yang*. Despite these known differences between these two species of ginseng, little is known about the effects of North American ginseng on cardiovascular responses, given that the majority of research in this area have used Asian ginseng as the intervention.

Therefore, the purpose of this study was to examine the effect of acute administration of North American, water-soluble ginseng on rat cardiac contractile function at 1 and 24 h post-ginseng treatment and to explore potential direct effects upon the heart. We hypothesized that acute administration of North American ginseng would have no effect on the contractile performance of the myocardium.

## MATERIALS AND METHODS

### PREPARATION OF GINSENG ROOT EXTRACT

Four year old North American ginseng roots collected in 2007 from five different farms in ON, Canada were provided by the Ontario Ginseng Growers Association. Dried ginseng root samples were shipped to Naturex Inc. (South Hackensack, NJ, USA) for extraction. In order to reduce variability final ginseng extracts from each farm were prepared individually and were then combined to produce composite extracts at the Ontario Ginseng Innovation and Research Consortium central facility (London, ON, Canada) which were used for the present study. A detailed methodology for aqueous extraction has been previously published elsewhere (Azike et al., 2011). Briefly, dried ginseng root samples were ground and soaked in water at 40°C for a duration of 5 h. The resultant solutions were filtered and excess solvent was removed by a rotary evaporator under vacuum at 45°C. The ginseng pools were combined and concentrated to yield a concentration of 60%. The concentrates were lyophilized with a freeze drier at –50°C under reduced pressure to produce ginseng aqueous extract in powder form.

### ANIMALS

All animals were cared for according to the Guiding Principle in the Care and Use of Animals. All experiments were approved by the University of Western Ontario council on animal care committee. Seventy-seven, adult (8-week-old) male Sprague–Dawley rats (Charles River Laboratories International Inc. Sherbrooke, QC, Canada) were used in the study. The animals were acclimated to a 12-hour day-night cycle, housed in rodent cages, and fed standard rodent chow. Rats were gavaged with water (control) or water-soluble American ginseng extract (300 mg/kg body weight). According to the NHPD Monograph of Health Canada^1^, the recommended maximum daily dose of American ginseng for humans is 12 gm of dry roots; and this is equivalent to 170 mg/kg of body weight. After taking consideration of the 40–50% yield of extract from ginseng root according to the our processing protocol, and the extrapolation factor of five to one used in translating human to rodent dosage of therapeutics (because of species-difference in metabolic rate), our current dosage used is relevant to what is consumed in humans. HR and developed pressure were measured 1 and 24 h after gavaging. For each of the electrocardiographic (ECG) and non-invasive measurement of BP experiments (see below), 24 animals were used. They were divided into a) control animals (*n* = 8) measured at both 1 and 24 h post-gavage with water and, b) experimental animals (*n* = 8) assessed at 1 and 24 h post-gavage with ginseng. Twenty-four animals were used in the Langendorff (LP) experiment (*n* = 6 each for 1 h control, 1 h ginseng, 24 h control, and 24 h ginseng groups) and five in the isolated myocyte experiment.

#### Electrocardiography

Three-lead ECGs were recorded while rats were under light anesthesia (isoflurane – 2% in 99% O_2_). All animals had their ECG’s recorded for only 10 min to avoid the side effects of prolonged anesthesia. Total time per animal for the complete procedure was no longer than 15 min. For this study, subcutaneous needle electrodes were used for ECG, as described previously (Tipton and Taylor, 1965). The ECG signal was recorded using a Honeywell ECG amplifier (Honeywell Electronics for Medicine, Edmonton, Canada). The analog electrocardiogram was sampled at 1000 Hz and recorded using a Powerlab 8/30 Data Acquisition system and analyzed using Labchart 7.0 pro software (ADInstruments, Colorado Springs, Colo, USA). The mean of 10 cycles was taken for the measurement of PR, QRS, and RR intervals.

### NON-INVASIVE MEASUREMENT OF BLOOD PRESSURE

Systolic and diastolic BPs were measured in conscious rats using a non-invasive computerized tail cuff system (CODA Non-Invasive BP Monitor, Kent Scientific Corporation, Torrington, CT, USA). Rats were conditioned to tail cuff instrumentation for three days before the experiment to minimize the effects of cuff inflation/deflation stress. BP analyses consisted of 15 tail cuff pressure acquisitions per run. Data for individual animals represent the average of at least five high-quality acquisitions. HRs were also recorded during these measurements.

### LANGENDORFF HEART PREPARATION

The rats were killed by decapitation and the hearts were immediately excised and immersed in ice-cold Krebs–Henseleit buffer producing an immediate cessation of contraction. Then the hearts were rapidly cannulated for retrograde aortic perfusion by a modified non-recirculating Langendorff technique (Su and Narayanan, 1993). The perfusion fluid was Krebs–Henseleit buffer (pH 7.4) consisting of (in mM): 120 NaC1, 4.63 KC1, 1.17 KH_2_PO_4_, 20 NaHCO_3_, 1.25 CaC1_2_, 1.20 MgCl_2_, and 8 mM glucose as substrate. The buffer was gassed with a 95% O_2_/5% CO_2_ mixture. The entire perfusion system was maintained at 37°C using a water-jacketed condenser attached to a circulator. Unpaced hearts were perfused at a constant flow rate of 9 ml/min. In a subset of 1 and 24 h control hearts (*n* = 4), control data was recorded initially and then successive doses of ginseng (25 and 250 μg/ml for 3 min each) were added. Cardiac function was determined under each condition and hearts were washed out and returned to baseline function (10–15 min) between these successive ginseng administrations.

To determine left ventricular function, a water-filled latex balloon was inserted into the left ventricle through the mitral valve. Left ventricular end-diastolic pressure (LVEDP) was adjusted to 5 mmHg by increasing the balloon volume with a micrometer-fitted syringe. Left ventricular pressures (LVEDP and developed pressure) were obtained with a pressure transducer (Statham Gould P23ID). The rate of pressure development (+dP/dt) and relaxation (-dP/dt) were obtained electronically using a Powerlab 8/30 Data Acquisition system and analyzed by Labchart 7.0 pro software (ADInstruments, Colorado Springs, Colorado, USA).

### ISOLATION OF CARDIAC MYOCYTES

Five additional rats were used for this part of the study. Isolation of adult rat ventricular myocytes was carried out using a method described by Kilic et al. (2009). In brief, the heart from each rat was removed and retrogradely perfused with a Ca^2^^+^-free buffer. After 5 min of perfusion, the heart was switched to a buffer containing collagenase (Worthington Biochemical, Lakewood, NJ, USA) and proteases. At the end of digestion, the heart was removed and the ventricles were minced into small pieces in stop buffer. The cell suspension was filtered through a nylon 210 size mesh and the extracellular Ca^2^^+^ concentration was increased in a stepwise fashion (up to 1 mM). The viability of cardiomyocytes was greater than 90% of the final cell population.

### FLUORESCENCE RECORDING OF [Ca^2^^+^]_i_

[Ca^2^^+^]_i_ was measured using fura-2 fluorescence from single cardiac myocytes as described previously (Weidema et al., 1997). Cells were loaded by incubation with 0.5 μM fura-2 acetoxymethylester (Molecular Probes) for 30 min at room temperature. The cells were continuously superfused with Sodium Ringer solution containing 1 mM CaCl_2_ and field stimulated with bipolar platinum electrodes, using a pulse duration of 1 ms and a frequency of 0.5 Hz, with voltage set at 60–80 V. Intracellular calcium fluorescence measurements were made using a dual-wavelength Deltascan spectrofluorimeter [Photon Technology International (PTI)]. Cells were illuminated by epifluorescence with alternating 345 and 380 nm light from a Xenon lamp and a Nikon Fluor 40× objective lens and recorded by photometer. The emission signal was filtered using a 510 nm bandpass filter. [Ca^2^^+^]_i_ was calculated from the ratio of the fluorescence intensities at 345 and 380 nm following correction for background.

### STATISTICAL ANALYSIS

Data are presented as means ± SEM. Statistical significance was assessed with either the Student’s *t*-test (to compare before and after ginseng treatment on Langendorff preparations at 25 and 250 μg/ml) or one-way and repeated measures ANOVA, followed by Tukey *post hoc* test (all other comparisons). *p *< 0.05 was taken as the level of significance.

## RESULTS

**FIGURE 1:**
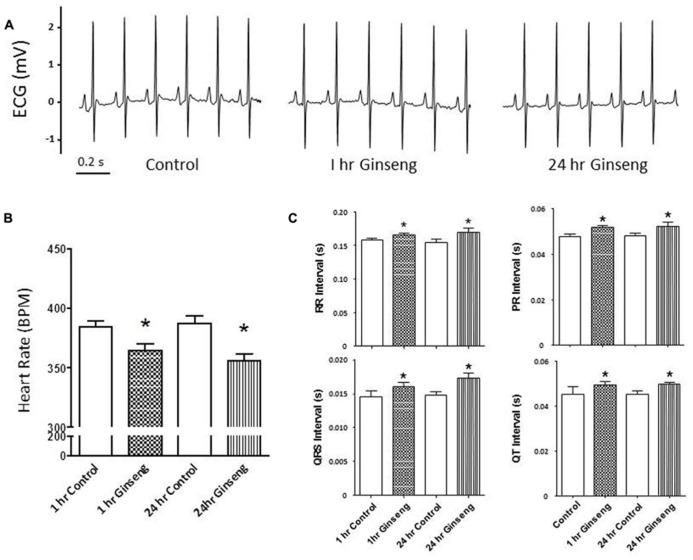
**Electrocardiogram data recorded from control animals and those treated with water-soluble ginseng (*n* = 8/group; 1 and 24 h after gavaging). (A)** Displays typical ECG tracings; **(B)** Displays typical HR responses; and **(C)** Shows that the RR, PR, QRS, and QT intervals. Data are presented as mean ± SE. *p < 0.05 compared to control.

**Figure 1** shows ECG data recorded from control rats and rats treated with water-soluble ginseng (1 and 24 h after gavaging). There was a group effect for HR so that it was lower in ginseng treated rats compared with control rats at both 1 and 24 h after ginseng gavage (*p* < 0.05; **Figure 1A**). HR did not differ between 1 and 24 h groups in either the control or the ginseng treated conditions, indicating no time-dependence (*p* < 0.05; **Figure 1A**). The RR, PR, QRS, and QT interval were consistently prolonged in ginseng treated rats compared with controls (*p* < 0.05; **Figure 1B**). These data are summarized in **Table 1**.

**Table 1:** ECG data from control and 1 and 24 h water ginseng treated rats.

	Heart rate (BPM)	RR interval (s)	PR interval (s)	QRS interval (s)	QT interval (s)
1 hr control	385 ± 6	0.158 ± 0.003	0.047 ± 0.001	0.015 ± 0.001	0.045 ± 0.003
1 hr ginseng	365 ± 5*	0.165 ± 0.003 *	0.052 ± 0.001*	0.017 ± 0.001*	0.05 ± 0.004*
24 hr control	388 ± 6	0.154 ± 0.004	0.048 ± 0.001	0.015 ± 0.001	0.045 ± 0.002
24 hr ginseng	356 ± 6*	0.169 ± 0.005 *	0.052 ± 0.002*	0.017 ± 0.001*	0.05 ± 0.005*

Data are expressed as mean ± SEM, *n* = 8 for control and ginseng treated group. *p < 0.05 vs. control. BPM, beats per minute.

**FIGURE 2:**
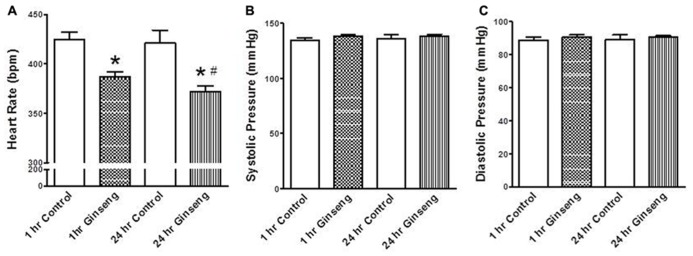
**Non-invasive measures of blood pressure recorded from control animals and those treated with water-soluble ginseng extract (*n* = 8/group; 1 and 24 h after gavaging). (A)** Shows the HR responses. **(B,C)** Display systolic and diastolic blood pressure, respectively. Data are presented as mean ± SE. *p < 0.05 compared to control; ^#^*p* < 0.05 compared to 1 h ginseng treated.

Non-invasive measurements of HR and BP are depicted in **Figure 2**. HR and developed pressure of the heart (removed from animals treated 1 and 24 h after gavage with ginseng) were also measured *ex vivo* via a Langendorff preparation. HR was lower in ginseng treated groups compared with control rats (1 h control, 424 ± 8 beats per minute (BPM); 1 h ginseng 387 ± 6 BPM; 24 h control: 421 ± 13 BPM, 24 h ginseng, 372 ± 7 BPM, *p* < 0.05, **Figure 2A**). In the ginseng treated group, HR was lower at 24 h compared to 1 h after gavaging (*p* < 0.05, **Figure 2A**). Systolic and diastolic pressures did not differ between the control and ginseng treated groups at any time point (*p* > 0.05; **Figures 2B,C**).

**FIGURE 3:**
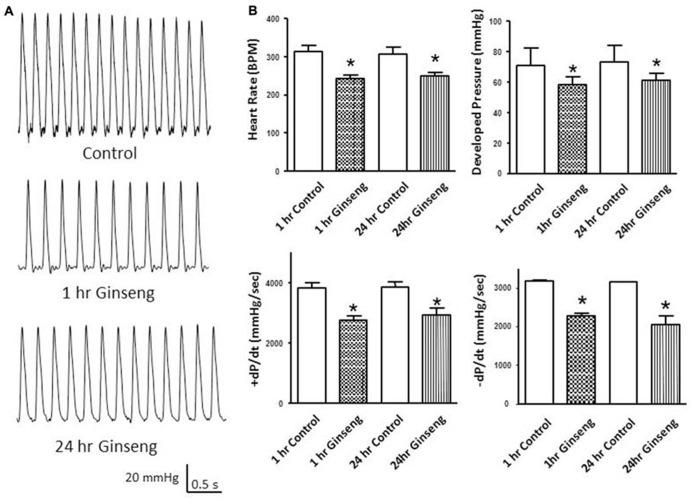
**Langendorff isolated-perfused heart data recorded from control and water-soluble ginseng extract treated rats (*n* = 6/group; 1 and 24 h after gavaging). (A)** Shows representative traces of developed pressure recorded from spontaneous beating hearts of control and ginseng treated rats. **(B)** Depicts the HR and developed pressure responses. Data are presented as mean ± SE. *p < 0.05 compared to control.

Heart rate obtained during the Langendorff preparation was also lower in hearts taken from ginseng treated compared to control rats (1 h control, 313 ± 16 bpm; 1 h ginseng, 243 ± 9 bpm; 24 h control, 308 ± 17 bpm; 24 h ginseng: 248 ± 10 bpm; *p* < 0.05; **Figure 3B**). Similarly, developed pressure and rate of change in pressure development (+dP/dt and –dP/dt) was reduced in the ginseng treated compared to the control animals (for developed force – 1 h control: 88 ± 6 mmHg; 1 h ginseng: 63 ± 5 mmHg; 24 h control, 89 ± 7 mmHg; 24 h ginseng, 61 ± 5 mmHg; *p* < 0.05; **Figure 3B**; for +dP/dt – 1 h control: 3823 ± 186 mmHg/s; 1 h ginseng: 2740 ± 153 mmHg/s; 24 h control, 3864 ± 201 mmHg/s; 24 h ginseng, 2922 ± 239 mmHg/s; *p* < 0.05; **Figure 3B**; and for –dP/dt -1 h control: 3183 ± 42 mmHg/s; 1 h ginseng: 2266 ± 79 mmHg/s; 24 h control, 3147 ± 35 mmHg/s; 24 h ginseng, 2057 ± 210 mmHg/s; *p* < 0.05; **Figure 3B**). However there was no main effect for time (i.e., 1 vs. 24 h) for either any of these variables. No difference was observed in the baseline coronary perfusion pressure in the constant flow perfused heart preparations: control 50 ± 3 mmHg, 1 h ginseng 49 ± 2 mmHg, 24 h ginseng 51 ± 3 mmHg.

**FIGURE 4:**
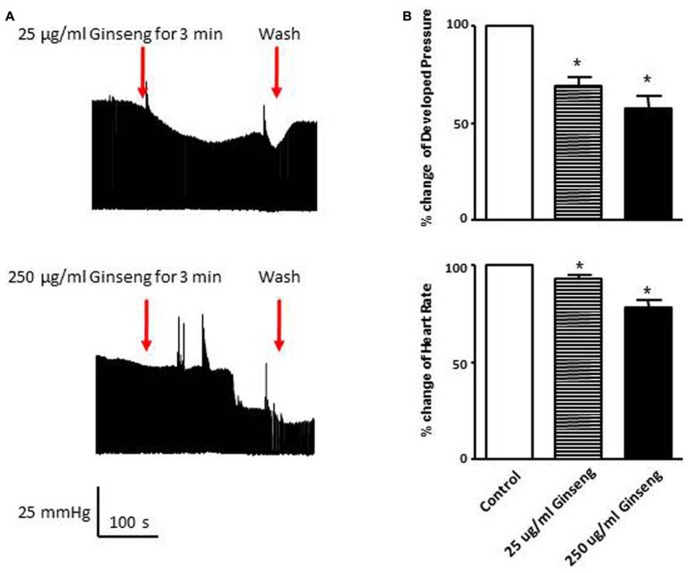
**Effect of direct administration of water-soluble ginseng extract (25 and 250 μg/ml) on developed pressure and heart rate of Langendorff isolated-perfused rat hearts (*n* = 4/group). (A)** Shows the representative traces of developed pressure recorded from spontaneous beating rat hearts after 25 and 250 μg/ml ginseng treatments. **(B)** Displays the percentage change in developed pressure and HR. Data are presented as mean ± SE. *p < 0.05 compared to control.

**FIGURE 5:**
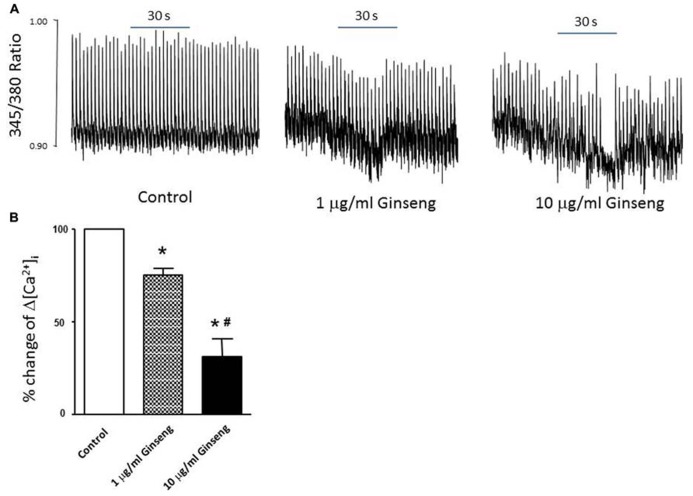
**Effect of water-soluble ginseng extract (1 and 10 μg/ml) on intracellular Ca^2^^+^ transient changes in rat ventricular myocytes. (A)** Typical experiments showing the effect of direct administration of vehicle control, 1 and 10 μg/ml ginseng for 30 s on intracellular Ca^2^^+^ transients. The fura-2 loaded myocyte was paced at 0.5 Hz (see Materials and Methods for details). **(B)** Concentration-dependent response of ginseng on intracellular Ca^2^^+^ transient changes. Δ[Ca^2^^+^]_i_ expressed as the percentage change of control. Data are presented as mean ± SE. *p < 0.05 compared to control; ^#^*p* < 0.05 compared to 1 μg/ml ginseng dose. *n* = 5 in each group.

In the subset of rats in which different doses of ginseng were added to the perfusate following collection of control data (i.e., not pre-treated with ginseng), there was a dose-dependent effect of ginseng on cardiac function. Developed pressure and HR were decreased in response to both 25 μg/ml (developed pressure was 69 ± 4% and HR was 93 ± 3% of control) and 250 μg/ml (developed pressure was 58 ± 7% and HR was 78 ± 7% of control), *p* < 0.05; **Figure 4**). For the perfused heart, we also noted that washout of the test compounds was on occasion accompanied by decrease in function before recovery of both HR and developed pressure (by 3–5 min post-washout) was seen. This was likely due to indirect effects associated with changes in the perfusate. We extended the whole heart study to examine Ca^2^^+^ transients in single isolated cardiomyocytes. Cells were loaded with the Ca^2^^+^-sensitive dye fura-2 and monitored by spectrophotometry. The 345/380 ratio reflects Ca^2^^+^ concentration. Water-soluble ginseng was applied focally by pressure ejection from a micropipette. Whereas vehicle control had no effect on [Ca^2^^+^]_i_ (**Figure 5A** at left), treatment with ginseng did cause a decrease in the [Ca^2^^+^]_i_. Ca^2^^+^ transients were progressively decreased in response to 1 μg/ml 75 ± 4% and 10 μg/ml 31 ± 10% ginseng (*p* < 0.05; **Figure 5B**). In addition to the decrease in the calcium transients, as shown in **Figure 5B**, there was a small decline in basal calcium levels amounting to ratio changes of 0.79 ± 0.06 for 1 μg/ml and 0.76 ± 0.04 for 10 μg/ml, for the periods of ginseng application.

## DISCUSSION

The goal of this study was to determine the effects of North American, water-soluble ginseng on cardiac contractile function using both *in vivo* and *ex vivo *models. The main finding was that acute administration of ginseng resulted in depressed cardiac contractile function, as evidenced by reductions in HR and developed pressure, which persisted up to 24 h. This acute reduction in cardiac contractile function appears to be intrinsic to the myocardium, as a direct inhibitory effect of North American ginseng was observed on spontaneously beating perfused hearts exposed to different concentrations of ginseng, and on intracellular calcium transients in freshly isolated cardiac myocytes.

### HEART RATE RESPONSE

Heart Rate was significantly reduced in response to treatment with ginseng. This held true for each of the different methods used to measure HR (ECG, ~6–8% decrease; tail cuff inflation, ~9–13%; Langendorff preparation, ~19–22%). This observation is in line with previous reports showing that, in humans, ginseng administration resulted in a significant reduction in HR (Kiesewetter et al., 1992; Zheng and Moritani, 2008). It is quite likely that the negative chronotropic effects of American ginseng occurs at all levels of the heart, including sinoatrial node, atrioventricular node conduction and cardiac muscle as ECG intervals reflecting these events were all similarly depressed (**Figure 1**; **Table 1**). The depressed HR response observed in the ginseng treated group might be related to some of the bioactive compounds of ginseng. The active ingredients of ginseng which lead to the alterations of heart function are not fully understood. In terms of the water-soluble North American ginseng utilized in this study, it contains not only ginsenoside Re (G-Re), but also other ginsenosides (Rb_1_, Re, Rc, Rd, Rg_1_, and Rb_2_) which might interact with each other. Moreover, high-performance liquid chromatography analysis of the aqueous ginseng extracts has shown that they are enriched in polysaccharides (Azike et al., 2011). Available studies have shown that ginseng polysaccharides are active components and have a number of pharmaceutical activities (including anti-tumor, antioxidant, and hypoglycemic activities (Konno et al., 1985; Shin et al., 2004; Luo and Fang, 2008). Unfortunately, limited information is currently available about the effects of ginseng polysaccharides on the heart. In contrast, it is clear that specific ginsenosides may influence cardiac function. For example, G-Re suppresses mechanical alternans recorded from human and cat atrial myocytes and feline Langendorff-perfused hearts (Wang et al., 2008). This may be a consequence of an increased open probablility of sarcoplasmic reticulum (SR) ryanodine receptors resulting in reduced SR calcium content (Wang et al., 2008) an event which could lead to the reduction in internal calcium of myocytes observed in the present study. The current observation of reduced calcium transients (**Figure 5**) further suggests that force generation by the ventricular myocytes may have been compromised upon the addition of ginseng and this would help explain the reduced developed pressure noted in the Langendorff preparation (**Figures 3** and 4). G-Re has previously been observed to decrease force and rate of contraction in a dose dependent manner in an atrial preparation from guinea pigs, possibly due to blockage of calcium channels (Jin and Liu, 1994; Jin, 1996).

Hence, at this point it is unclear whether differences in profile or content of ginsenosides or polysaccharides are responsible for the observed effects on HR. Given these observations, a systematic examination of the different ginseng components and indeed type of ginseng (North American vs. Asian) is warranted.

### CHANGES IN DEVELOPED PRESSURE

In this study, systemic BP was measured *in vivo* and developed pressure of the heart was assessed* ex vivo*. Although the non-invasive *in vivo* measurements suggested no alterations in BP in response to ginseng administration, the *ex vivo* responses derived from the Langendorff isolated-perfused hearts indicated a significant reduction in the left ventricle developed pressure. Although these results may appear contradictory, it is important to note that during the *in vivo* measurements, the ginseng treated animals remained under the influence of neural and humoral regulation. This is important because any changes observed during the *ex vivo* preparation must be related to intrinsic properties of the myocardium. The lack of effect of ginseng administration on BP is a common observation in the live animal (Moey et al., 2012; Pan et al., 2012) and****can possibly be accounted for by compensatory mechanisms. Indeed, as cardiac output was not measured *in vivo*, the observed maintenance of BP (**Figure 1**) suggests that stroke volume may have been increased, so that cardiac output was maintained,**possibly as a consequence of the Frank-Starling mechanism, enhanced sympathetic drive or a combination of both. Similarly, enhanced sympathetic drive could lead to peripheral adaptations in the vasculature which could maintain BP *in vivo*. In the absence of these compensatory adaptations in the live animal, ginseng appears to depress both HR and contractile function of the isolated heart.

### EVIDENCE OF ACUTE REDUCTION IN CARDIAC CONTRACTILE FUNCTION APPEARS TO BE INTRINSIC TO THE MYOCARDIUM

In order to further examine the direct effect of North American ginseng on rat cardiac contractile function, spontaneously beating perfused hearts were examined at varying concentrations of water-soluble ginseng by using the Langendorff preparation. HR and developed pressure were significantly decreased when ginseng was administered but the changes in HR and developed pressure were greater as the ginseng concentration was increased from 25 to 250 μg/ml. This suggests not only that administration of North American ginseng has an effect on the rat cardiac contractile function, but also that this response might occur in a dose (concentration)-dependent manner.

To further understand the factors underlying the decrease in developed pressure of the heart, changes in intracellular Ca^2^^+^ were evaluated in isolated myocytes. After direct treatment with ginseng, decreased intracellular Ca^2^^+^ transient changes were observed using the fluorescent indicator fura-2 in freshly isolated cardiac myocytes. A small decline in basal calcium levels amounting to ratio changes of 0.79 ± 0.06 for 1 μg/ml and 0.76 ± 0.04 for 10 μg/ml, for the periods of ginseng application was also noted. As noted above, previous studies have shown that the ginsenosides Re and/or Rb1 suppressed intracellular Ca^2^^+^ transients in cat, human and rat myocytes (Scott et al., 2001; Wang et al., 2008); however, the mechanisms regulating this inhibitory effect of ginsenosides remain to be fully elucidated. It has been suggested that the inhibitory effect might be related to blocking both the probability and duration of opening of the plasma membrane L-type Ca^2^^+^ channel (Zhang et al., 1994; Zhong et al., 1995). Alternatively, enhanced opening of ryanodine receptors localized at the SR (Wang et al., 2008) or stimulation of nitric oxide production (Gillis, 1997; Scott et al., 2001) have been also proposed as potential mechanisms controlling this reduction in intracellular Ca^2^^+^. Interestingly, while these experiments on isolated myocytes may help explain the decline in developed pressure with the Langendorff preparation, they cannot account for the HR changes observed in the *in vivo* (**Figure 1**) and *ex vivo* (**Figures 3** and 4) experiments, as we paced the myocytes and did not specifically isolate pacemaker cells. Overall, data from the present study support the idea that administration of North American ginseng (*P. quinquefolius*) has important effects on cardiac contractile function and suggest that those effects are the result of changes in intrinsic properties of the myocardium, likely associated with calcium handling.

## CONCLUSION

In conclusion, this study did not support the hypothesis that acute administration of North American ginseng would have no effect on cardiac function. Rather it demonstrated that acute administration depresses cardiac contractile function in the rat as early as 1 h and up to at least 24 h after its administration. This acute reduction in cardiac contractile function appears to be dose-dependent and to rely on mechanisms intrinsic to the myocardium.

## Conflict of Interest Statement

The authors declare that the research was conducted in the absence of any commercial or financial relationships that could be construed as a potential conflict of interest.

## Footnotes

1
